# Beyond buzzing: mosquito watching stimulates malaria bednet use—a household-based cluster-randomized controlled assessor blind educational trial

**DOI:** 10.1038/emi.2013.67

**Published:** 2013-10-09

**Authors:** Tomonori Hoshi, Paul Martin Banda, Dylo Foster Pemba, Toshihiko Sunahara, Noboru Minakawa

**Affiliations:** 1 Graduate School of International Health Development, Nagasaki University, Nagasaki 852-8523, Japan; 2 Institute of Tropical Medicine (NEKKEN), Nagasaki University, Nagasaki 852-8523, Japan; 3 Independent Researcher in Liphuphwe, P.O. Box 180, Dedza, Malawi; 4 Department of Biology, Chancellor College, The University of Malawi, P.O. Box 280, Zomba, Malawi

**Keywords:** bednet, behavior change, health education, malaria, mosquito entomology

## Abstract

Malaria remains a severe health problem in Sub-Saharan Africa, with approximately one million deaths and 365 million cases each year. In terms of malaria control, insecticide-treated bednets are an effective tool, and many organizations have distributed free or highly subsidized bednets in malaria endemic areas. Nevertheless, some recipients do not use bednets because of social, environmental or cultural factors. Making vulnerable populations aware of the presence of mosquitoes may improve bednet use among people owning but not using a bednet. We hypothesized that showing freshly collected mosquitoes from the vicinity could improve bednet use in households owning but not using bednets. To test this hypothesis, we applied a household-based cluster-randomized controlled assessor blind educational trial. Indirect observation of mosquitoes, via educational leaflets, produced no change in bednet use, while showing freshly captured mosquitoes led to a 13-fold increase in bednet use. Our results suggest that direct observation of freshly captured mosquitoes can encourage bednet use and may potentially improve effective bednet coverage for malaria control and elimination.

## INTRODUCTION

Bites are independent of mosquito buzzing,^1^ yet anybody hearing mosquito buzzing will unequivocally be concerned about being bitten (or acquiring an infectious disease). The process by which mosquito buzzing becomes synonymous with bites relies on the realization that mosquito buzzing is often followed by bites. When people hear the sound of buzzing, it is only natural to seek defense against mosquito bites.^2,3,4^ Insecticidal treated bednets (ITNs), especially long-lasting insecticide-treated nets, are effective in preventing malaria and widely used in developing countries.^5,6,7^ ITNs contributed to the dramatic reduction of malaria deaths and cases over the past 10 years.^8,9,10,11^ However, many residents still do not use ITNs even though they possess them.^12,13^ In other words, ITN ownership alone cannot solve the problem of ITN use. For example, some studies have reported that ITNs are used for fishing or to protect agricultural crops instead of for personal protection.^14,15^ Furthermore, housing structural factors can hamper ITN use.^16^ Hence, social, behavioral and/or housing quality often impede ITN use. To overcome these difficulties and enhance ITN use, educational programs aimed at improving the understanding of malaria infection risk and prevention could be a pillar for long-term malaria suppression.

Effective education can alter human behavior. In addition, visual tools may prove more effective at transferring knowledge than the abstract presentation of ideas.^17^ For example, studies on condom for preventing sexually transmitted diseases have shown that video-based education improves condom use in sexually active populations.^18,19^ Likewise, researchers have explored suitable visual approaches to improve ITN use.^20,21^

Here, we present results from a household-based cluster-randomized controlled assessor blind educational trial to encourage ITN use. In our study, illustrated leaflets, a traditional visual tool, were compared with the presentation of freshly collected mosquitoes, an innovative educational tool to encourage ITN use. We hypothesized that freshly caught mosquitoes have a deep visual impact on how populations perceive the threat of mosquitoes. The presentation of freshly caught mosquitoes can demonstrate the presence of mosquitoes and therefore encourage ITN use for people owning but not using ITNs. Our data showed that people who were shown freshly collected mosquitoes were about 13 times more likely to use ITNs than those who were shown an educational leaflet or received no further information on malaria. Our results suggest that the realization of the presence of malaria vectors by direct observation can encourage ITN use and potentially improve effective ITN coverage for malaria control and elimination. Hence, the display of freshly collected mosquitoes could potentially improve the effectiveness of ITN coverage for malaria control or elimination by increasing ITN use among people owning but not using ITNs.

## MATERIALS AND METHODS

### Pre-intervention stage

**Figure 1:**
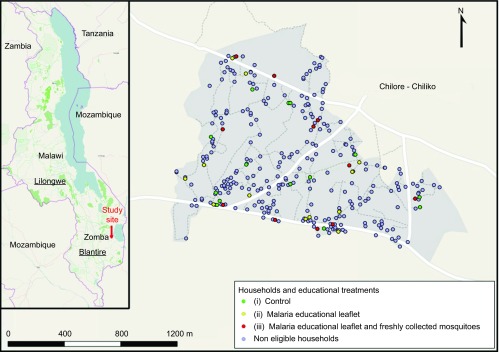
Chilore–Chiliko, Zomba district, Malawi. The box shows the location of study site within Malawi.

**Figure 2:**
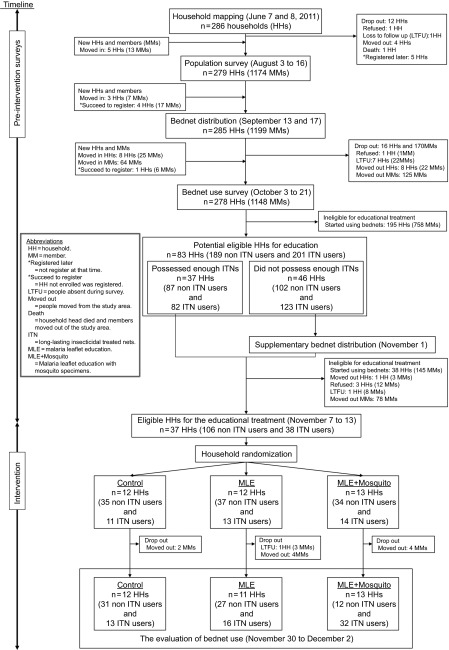
Flowchart of participants though the educational trial and timeline.

The Chilore–Chiliko (Figure 1) community is located approximately nine km west of Lake Chilwa, Malawi and stands at 650 m above sea level. The majority of the study population consisted of the Nyanja tribe. This area has a dry season from May to October, and a wet season from November to April.^22^ The temperature is relatively cool in the Sub-Saharan African context ranging from 14 °C to 21 °C during the night time. According to local health center records, malaria is endemic in this community without marked seasonal fluctuations, and 351 malaria cases were recorded in 2012. Our study was conducted between 7 June and 2 December 2011 (Figure 2). All households, 286 in total, were mapped in the community using global positioning system units (Garmin GPSMAP 62s and 60CSx; Garmin Ltd, KS, USA). A baseline population survey was conducted to census the population, and 1199 residents were registered. Based on this population size, we estimated that 848 Long Lasting ITNs (Olyset Net; Sumitomo Chemical Co. Ltd, Tokyo, Japan) were necessary to cover all household members in our study site, i.e., to ensure full coverage.^12,23,24^ The full coverage estimation was obtained by adding household coverage estimates. For each household, a coverage estimate was obtained by picking the largest number between: (i) the number of sleeping places and (ii) the rounded number of household members divided by two, where the number two is chosen as denominator under the assumption that one ITN can be shared by two people. In the middle of September, we distributed free ITNs, and two months after ITN distribution, we conducted an ITN use evaluation survey. We asked each household head which household members slept the night before the survey in the house and which of those members slept under an ITN during the previous night, a standard method to assess ITN use.^9,12,16,23^ All surveys and educational materials were translated into Chichewa, the local language in our study area. Details about the number of enrolled participants, and activity chronology, are presented in Figure 2, pre-intervention stage.

### The randomized controlled educational trial

Based on the ITN use evaluation survey, we found 83 potentially eligible households, which had at least one member who had not started using ITNs (Figure 2, intervention). In 46 households, we needed to supply additional ITNs to cover all householders, while the remaining 37 households had sufficient ITNs. After this supplementary ITN distribution, only 37 households had ITNs that were not being used. These households were enrolled in our randomized controlled educational intervention, since our goal was to enroll people that owned but did not use ITNs. The intervention began by surveying household heads regarding their knowledge of malaria transmission. The questionnaire contained 16 questions and the sum of correct answers was used to develop a malaria knowledge index. This index ranged from 0 to 16. The day of the survey, we also collected mosquitoes using Centers for Disease Control and Prevention miniature light traps (model 512; John W. Hock Company, FL, USA). Traps ran from 5:00 p.m. to 5:00 a.m. at each household main bedroom.

The three arms in our household-based cluster-randomized controlled intervention were: (i) control: household heads did not receive any education on malaria; (ii) malaria educational leaflet: household heads received a leaflet with pictures and cartoons, issued by Total S.A., Courbevoie, France (Supplementary Figure S1). This leaflet was supplemented by additional entomological information on mosquito entomology (Supplementary Figure S2); and (iii) malaria educational leaflet and freshly collected mosquitoes: household heads received the educational leaflets and were shown freshly collected, live and buzzing, mosquitoes from inside their houses.

Malaria leaflet (Supplementary Figure S1) for arms (ii) and (iii) were delivered when Centers for Disease Control and Prevention traps were retrieved from the households, together with the additional entomological leaflet, [arm (ii)], and the freshly collected mosquitoes, [arm (iii)]. The time spent going through the malaria leaflet and showing mosquitoes (leaflet or fresh samples) was around 15 min in total, with negligible variability. To prevent any interviewer bias, the staff members performing the malaria knowledge surveys were blind to the arm assignment of each household. Moreover, staff in charge of the educational training did not have access to household heads' malaria knowledge scores prior to the intervention and did not participate in the post-intervention malaria knowledge and ITN use evaluation survey. Similarly, the same staff never performed pre- and post-intervention surveys with the same household heads. Furthermore, prior to the intervention, all the interviewers were evaluated for the accuracy of their malaria knowledge scoring and ITN use evaluation skills, and we found no differences in their performance. Further details on activity chronology are presented in Figure 2, intervention.

### Ethical clearance

This study was approved by the ethical committee of Nagasaki University, Japan and by Chancellor College, The University of Malawi, Zomba, Malawi. All participants provided informed consent to collect mosquitoes inside the houses and to participate in the intervention.

### Data analysis

Enrolled participants were classified by age into three categories: children under 5 (CU5), 5–15 years (children) and above 15 years (adults). The educational level was also divided into three categories: no education, if a person had never attended school; primary school, for people that finished or dropped out from school, while they were in primary (a.k.a., ‘elementary') school; above primary, for people with education beyond primary. We used Fisher's exact test to test differences in sex, age structure and education level among the three treatment arms. We also performed one way analysis of variances (ANOVAs) to test for differences in malaria knowledge and mosquito density among the treatment arms. For the mosquito density, data were normalized via a log-transformation. Moran's I index was computed on the residuals of each ANOVA to test for any possible spatial autocorrelation that could hamper the validity of our inferences.^25^

We analyzed post-intervention ITN use with logistic generalized estimating equation (GEE) models. This method only requires a link function and the variance of the observations to allow sound inference on data that are not fully independent.^26^ In our study, the lack of independence of individuals belonging to the same household constrained the use of simpler regression strategies.^25^ For the models, we assumed independence in the correlation structure, provided the ability of GEE to obtain consistent estimates for the fixed effects even when the correlation structure is incorrect.^26^ However, for the inference, we used a sandwich estimator to obtain robust standard errors, provided that the naive standard errors are appropriate only when the correlation structure is correct.^26^ That GEE data are modeled at the population level, i.e., GEE parameter estimates represent the effect of a predictor averaged across all individuals in a population.^25^

To select the factors explaining the ITN use after the education, we used a strategy of backward stepwise model selection based on the quasi-likelihood information criterion.^27^ Briefly, a full model is simplified until a minimum quasi-likelihood information criterion is obtained. Our full model considered the following five factors: educational intervention, mosquito density, age, sex and educational level.

We analyzed all data using R (version 2.15.2, 32 bit). A *P* value of less than or equal to 0.05 was considered to be significant for all the analyses.

## RESULTS

We conducted our study in the Chilore–Chiliko community of Zomba district, Malawi (Figure 1). This community consisted of 286 households and 1199 inhabitants when we began the study. After several surveys (Figure 2), we determined that 36 households were eligible targets for our intervention, as they had a similar number of residents that had not used ITNs (Figure 2). These 36 households had 144 residents, with 74% not using ITNs, i.e., 106 residents, of which 96 were followed from the beginning of our study (Figure 2). For the intervention, households were randomly divided into three arms. Among the arms, differences in sex ratio (*P*=0.28), age (*P*=0.56) and educational level (*P*=0.37) were not significant according to a Fisher's exact test (Supplementary Table S1). The three arms were: (i) control, which received no additional information on malaria transmission; (ii) malaria educational leaflet, which received leaflets on malaria transmission and mosquito entomology; and (iii) malaria educational leaflet and freshly collected mosquitoes, which received the malaria transmission leaflet and were shown freshly collected mosquitoes from the household.

After the educational intervention, ITN use dramatically increased in members from households which were shown freshly collected mosquitoes, [arm (iii)], in sharp contrast with the other two arms (Table 1). When effects of the educational intervention on ITN use were evaluated, the best GEE model included two factors: educational treatment and age (Table 2). Sex, educational level and mosquito density were excluded after a model selection (Supplementary Table S2). People shown live and buzzing mosquitoes were 13.01 times more likely to use ITNs compared with the control (*P*=0.004; Table 2). The arm shown only the leaflet, [arm (ii)], was not significantly different from the control arm. Children aged 5–15 years were 90% times less likely to use ITNs (*P*=0.001; Table 2). Differences in the malaria knowledge score across three arms (Supplementary Figure S3) were not significant according to ANOVA: pre- (*F*(2,33)=1.20, *P*=0.31) and post-intervention (*F*(2,33)=1.18, *P*=0.32), which supports that the possibility of interviewee bias was minimized. Mosquito density (Supplementary Figure S4) was also similar among the three arms (*F*(2,33)=0.37, *P*=0.69). All the ANOVAs assumptions about the general linear model were met. Data were spatially independent, therefore, ensuring a statistical inference (Supplementary Figure S5).

## DISCUSSION

Our results clearly indicate that malaria education using live and buzzing mosquitoes dramatically increased ITN use as compared with the control arm and education using only malaria leaflets (Supplementary Table 2). These findings correspond to the mathematical models, which have demonstrated that mosquito awareness promotes community intervention to eliminate mosquito breeding sites.^3^ Moreover, studies have revealed that higher mosquito density enhances ITN use in malaria endemic areas.^12,24,28^ These phenomena show that people may become aware of mosquitoes around them through visual and/or auditory recognition (or an itchy feeling). In our study, we attempt to accelerate this process of mosquito realization in order to improve ITN use. In short, educational programs that engage sensory perception by showing live and buzzing mosquitoes can make a remarkable difference in ITN use when compared to traditional leaflet based education.

We also uncovered that ITN use among children aged 5–15 years was not promoted (Table 2). One explanation for this phenomenon is that these children did not observe freshly collected mosquitoes. Alternatively, they might have been ignorant of malaria risks, or they may have not been able to deploy ITNs because of sleeping arrangements and/or a lack of suitable locations to hang bednets.^16,29^ Nevertheless, CU5 were protected by ITNs following the use of live, buzzing mosquitoes as educational aids.

In order to make our educational intervention applicable to a broader population, several practical modifications might be necessary. For example, the mosquito collection method using Centers for Disease Control and Prevention light traps might be unrealistic for education in the field; hence, an alternative method for mosquito collection needs to be explored.^1^ Moreover, although health educators need basic malaria and mosquito biological knowledge,^20^ we assume that live mosquitoes are easy to capture in malaria endemic areas where ITNs are available. In addition, researchers should attempt to ascertain the long-term impacts of mosquito watching education on ITN use. Regrettably, such insights are beyond the scope of this study. Furthermore, it must be noted that demonstrating live and buzzing mosquitoes was not a perfect solution for increasing ITN usage. For reasons our study has yet to ascertain, some residents chose not to use ITNs even after observing freshly captured mosquitoes, which might be because of personal preferences or other factors. Additional research could be undertaken to understand why participants did not use ITNs even after being shown freshly captured mosquitoes.

In summary, mosquitoes are everywhere in malaria endemic areas, and we revealed that recognition of mosquitoes drives ITN usage. We therefore believe that tailored education based on mosquito watching can ensure higher ITN use in a broader population if there are sufficient ITNs. This unique strategy to improve ITN use could possibly help achieve effective ITN coverage for malaria control and elimination,^30^ and we believe that it can be incorporated into routine vector surveillance. In addition, we assume that the strategy of showing live mosquitoes can be useful not only in communities, but also in schools.

## Figures and Tables

**Table 1:** Summary of enrolled households and members in each educational arm.

Arm	Number of households	Total number of people	People/household (mean±S.E.^a^)	People under ITNs after the intervention (%)
Control	12	33	2.75±1.66	4 (15.4%)
Leaflet	11	32	2.91±2.02	5 (19.2%)
Leaflet+mosquito	13	31	2.39±1.56	17 (65.4%)

a Standard error.

**Table 2:** Parameters estimates for a logistic generalized estimating equation model. The model considers the impact of educational treatment and age on bednet use.

Factor	COR^a^	(95% CI^c^)	Estimate	Robust S.E.^d^	Robust *Z*	AOR^b^	(95% CI^c^)	Estimate	Robust S.E.^d^	Robust *Z*
Control–CU5 (<5)	1	—	−1.98 to −0.41	0.66–0.56	−3.01 to −0.72	1	—	−0.95	0.84	−1.13
Leaflet+mosquito	8.80	(1.42–54.31)	0.73	0.93	2.34	13.01	(2.22–76.33)	2.57	0.90	2.84
Leaflet	1.34	(0.21–8.57)	0.66	0.95	0.31	1.10	(0.16–7.46)	0.09	0.98	0.09
Children (5–15 years)	0.23	(0.06–0.98)	−1.44	0.72	−1.99	0.10	(0.02–0.40)	−2.34	0.72	−3.24
Adults (>15 years)	0.91	(0.31–2.67)	−0.09	0.55	−0.17	0.56	(0.15–2.03)	−0.58	0.66	−0.89

a Crude odds ratio;

b Adjusted odds ratio;

c Confidence interval;

d Standard error.
