# Supplementary material for: Beyond buzzing: mosquito watching stimulates malaria bednet use—a household-based cluster-randomized controlled assessor blind educational trial
Source: Emerg Microbes Infect. 2013 Oct 9;2(10):e67–. doi: 10.1038/emi.2013.67 (PMC3826067; doi:10.1038/emi.2013.67)
Supplement: Supplementary information Figure S5 [file emi201367x1.pdf]

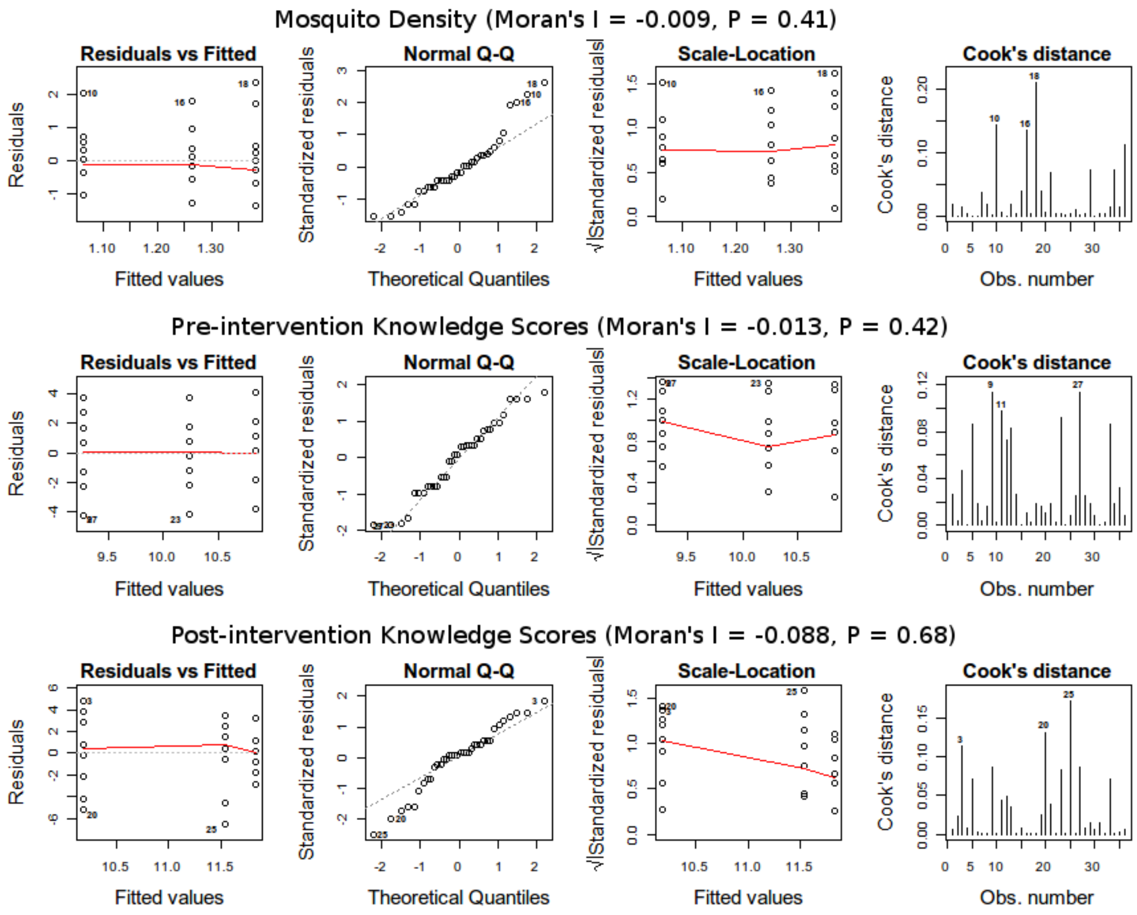

Supplementary Figure S5 ANOVA diagnostic plots and Moran's I for mosquito density, pre- and post- intervention malaria knowledge scores.
