# Supplementary material for: Beyond buzzing: mosquito watching stimulates malaria bednet use—a household-based cluster-randomized controlled assessor blind educational trial
Source: Emerg Microbes Infect. 2013 Oct 9;2(10):e67–. doi: 10.1038/emi.2013.67 (PMC3826067; doi:10.1038/emi.2013.67)
Supplement: Supplementary information Table S1 [file emi201367x2.doc]

**Supplementary Table S1** Baseline characteristics of participants among 3 treatment arms. For each of the 3 treatment arms, the number and percentage of participants with each characteristic is presented.

| Arm | Sex |  |  |  | Age |  |  |  |  |  | Education |  |  |  |  |  |
| --- | --- | --- | --- | --- | --- | --- | --- | --- | --- | --- | --- | --- | --- | --- | --- | --- |
|  | Male | (%) | Female | (%) | CU5 (<5) | (%) | Child (5-15) | (%) | Adult (>15) | (%) | No education | (%) | Primary school | (%) | Above primary | (%) |
| Control (n = 33) | 17 | (51.5) | 16 | (48.5) | 4 | (12.1) | 16 | (48.5) | 13 | (39.4) | 12 | (36.4) | 20 | (60.6) | 1 | (3.0) |
| Leaflet (n = 32) | 13 | (40.6) | 19 | (59.4) | 8 | (25.0) | 13 | (40.6) | 11 | (34.4) | 9 | (28.1) | 23 | (71.9) | 0 | (0.0) |
| Leaflet + Mosquito (n = 31) | 19 | (61.3) | 12 | (38.7) | 3 | (9.7) | 15 | (48.4) | 13 | (41.9) | 6 | (19.4) | 23 | (74.2) | 2 | (6.5) |
