# Supplementary material for: Beyond buzzing: mosquito watching stimulates malaria bednet use—a household-based cluster-randomized controlled assessor blind educational trial
Source: Emerg Microbes Infect. 2013 Oct 9;2(10):e67–. doi: 10.1038/emi.2013.67 (PMC3826067; doi:10.1038/emi.2013.67)
Supplement: Supplementary information Table S2 [file emi201367x3.doc]

**Supplementary Table S2** Logistic generalized estimating equation (GEE) model selection for factors associated with insecticide treated bednet use following the educational intervention. Each row presents the factors for each model. Individuals were grouped according to the household they belonged. QIC is the quasi-likelihood information criterion. Δ represents the difference with respect to the minimum value for QIG. **Minimum QIC is bolded**.

| Factors | QIC | ΔQIC |
| --- | --- | --- |
| Educational intervention, Age, Sex, Educational level, Mosquito density | 107.76 | 8 |
| Educational intervention, Age, Sex, Educational level | 105.84 | 6.08 |
| Educational intervention, Age, Educational level, Mosquito density | 106.69 | 6.93 |
| Educational intervention, Sex, Educational level, Mosquito density | 115.68 | 15.92 |
| Educational intervention, Age, Sex, Mosquito density | 103.74 | 3.98 |
| Age, Sex, Educational level, Mosquito density | 123.35 | 23.59 |
| Educational intervention, Age, Sex | 101.03 | 1.27 |
| Educational intervention, Age, Mosquito density | 102.48 | 2.72 |
| Educational intervention, Age, Educational level | 104.66 | 4.9 |
| Educational intervention, Sex, Educational level | 113.87 | 14.11 |
| Educational intervention, Sex, Mosquito density | 111.99 | 12.23 |
| Educational intervention, Educational level, Mosquito density | 114.67 | 14.91 |
| Age, Educational level, Mosquito density | 122.24 | 22.48 |
| Sex, Educational level, Mosquito density | 127.29 | 27.53 |
| **Educational intervention, Age** | **99.76** | **0** |
| Educational intervention, Educational level | 112.88 | 13.12 |
| Educational intervention, Mosquito density | 110.93 | 11.17 |
| Educational intervention, Sex | 109.38 | 9.62 |
| Age, Sex | 116.03 | 16.27 |
| Age, Educational level | 118.60 | 18.84 |
| Age, Mosquito density | 118.62 | 18.86 |
| Educational intervention | 108.32 | 8.56 |
| Age | 114.76 | 15 |
| Sex | 119.64 | 19.88 |
| Educational level | 121.78 | 22.02 |
| Mosquito density | 122.55 | 22.79 |
