# Supplementary material for: Beyond buzzing: mosquito watching stimulates malaria bednet use—a household-based cluster-randomized controlled assessor blind educational trial
Source: Emerg Microbes Infect. 2013 Oct 9;2(10):e67–. doi: 10.1038/emi.2013.67 (PMC3826067; doi:10.1038/emi.2013.67)
Supplement: Supplementary information Figure S2 [file emi201367x5.pdf]

Mosquitoes have long mouths and long legs.

Mosquito identification

Only female mosquitoes can take blood meals. Male mosquitoes have hairier antennae.

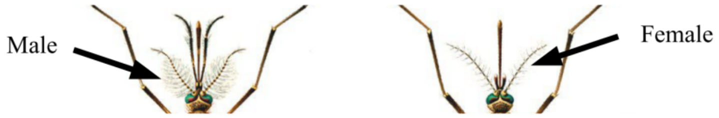

Below, you can see the two most common mosquitoes in this area. Both species are known to take blood meals at night. However, *Culex* mosquitoes do not transmit malaria.

Anopheles mosquito	*Culex* mosquito
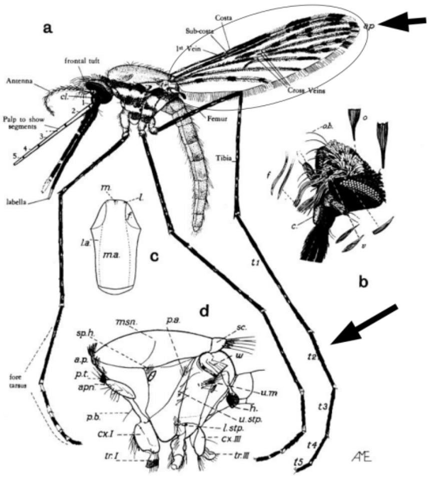 [The Anophelinae of Africa South of the Sahara (Ethiopian zoo-geographical region)]	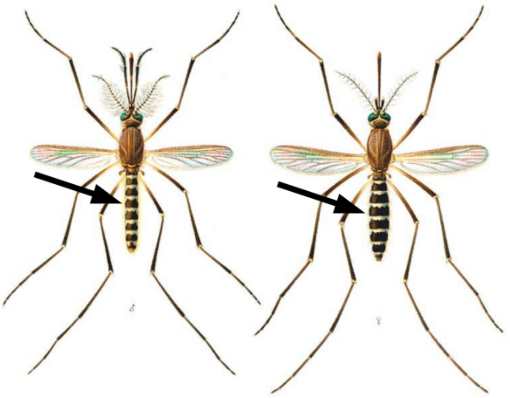 [http://en.wikipedia.org/wiki/Culex]
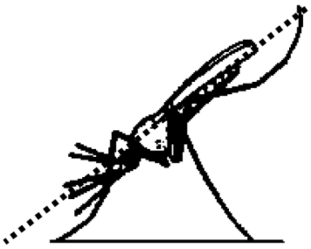	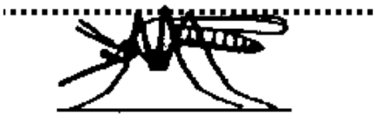 [http://www.msic.med.osaka-cu.ac.jp/citilec/97no13/top.html]

We found *Anopheles* mosquitoes around this village. *Anopheles* mosquitoes carry the malaria parasite and bite victims at night.

Supplementary Figure S2 Mosquito entomological leaflet.
